# Implementation of multifaceted diagnostic stewardship for *Clostridioides difficile* infection during the COVID-19 pandemic at a small Japanese hospital

**DOI:** 10.1017/ash.2024.93

**Published:** 2024-06-04

**Authors:** Yasuhiro Sasaki, Masataka Yano, Ayumi Umehara, Yasuaki Tagashira

**Affiliations:** 1 Department of Infection Control, Tama-Nambu Chiiki Hospital, Tokyo, Japan; 2 Department of Infectious Diseases, Tokyo Medical and Dental University (TMDU), Tokyo, Japan

## Abstract

**Objective:**

*Clostridioides difficile* infection (CDI) is a common, healthcare-associated infection. However, in Japan, testing for CDI is infrequent, suggesting that its incidence may be underestimated. This study aimed to examine the implementation of a multifaceted, diagnostic stewardship (DS) for CDI in a small Japanese hospital during the coronavirus 2019 pandemic.

**Design:**

Before-after study.

**Setting:**

A small Japanese community hospital.

**Participants:**

Healthcare workers including physicians, nurses, and pharmacists.

**Interventions:**

A multifaceted intervention including (1) the addition of CD testing criteria to the hospital guidelines; (2) provision of a tutorial on CD testing to physicians, nurses, and pharmacists; (3) assessment by clinical pharmacists and nurses of the need for CD testing in patients with nosocomial diarrhea and issuance of recommendations for CD testing to physicians; (4) reporting of data on the CD testing rate and CDI incidence in the study center.

**Results:**

The CD testing rate increased before the pandemic (+0.16/10,000 patient-days (PD); *P* = .28), decreased significantly during the pandemic (−0.79/10,000 PD; *P* = .02), and then increased significantly immediately after the implementation of the intervention (+29.6/10,000 PD; *P* < .01). Similarly, the CDI incidence increased significantly before the pandemic (+0.26/10,000 PD; *P* = .02) and decreased significantly during the pandemic (−0.49/10,000 PD; *P* = .01). Implementation of the intervention resulted in an immediate and significant increase in the CDI incidence (+6.2/10,000 PD; *P* < .01).

**Conclusion:**

Multifaceted DS involving multidisciplinary specialists was effective in improving CD testing, suggesting that appropriate testing can contribute to diagnosing CDI accurately.

## Introduction

*Clostridioides difficile* (CD) is the most commonly isolated pathogen in hospitals, and CD infection (CDI) is one of the most important, healthcare-associated infections.^1^ Therefore, it is important to diagnose and treat CDI appropriately. However, rather than the widely used blood and urine cultures, specific tests are needed to diagnose CDI. However, the decision to test often depends on the physician’s awareness of the importance of CDI as a healthcare-associated infection.

Previous, retrospective cohort studies^2–6^ in Japan reported the CD testing rate to be 24.5–44.0 per 10,000 patient-days (PD) and the CDI incidence to be 0.8–3.11/10,000 PD, demonstrating that the CD testing rate and CDI incidence varied widely among institutions. On the other hand, a multicentric, prospective study in Japan reported the CD testing rate to be 30.4/10,000 PD and the CDI incidence to be 7.4/10,000 PD.^7^ Furthermore, a systematic review reported a CDI incidence of 8.3/10,000 PD.^8^ The current CDI incidence in Japan may be underestimated due to the lack of appropriate testing. During the coronavirus disease 2019 (COVID-19) pandemic, the CDI incidence declined in the United States. Although the use of personal protective equipment and compliance with hand hygiene were cited as possible reasons for this decline,^9^ diarrhea is a symptom of CDI as well as of COVID-19,^10^ making it very difficult to determine its cause and leading to CDI possibly being overlooked in patients with COVID-19.^11^

The optimal CD testing rate is still unknown. The incidence of asymptomatic carriers in acute care hospitals is reportedly as high as 3%–26%, indicating an over-testing of asymptomatic patients,^12^ which can lead to unnecessary treatment. Appropriate testing can prevent overtreatment and enhance antimicrobial stewardship.^13^

Furthermore, diagnostic stewardship (DS) may conduce to appropriate antimicrobial use if a multidisciplinary team is involved in its implementation.^14^ The current guidelines state that unformed stools are an indication for testing but that solid stools are not^12^; thus, it is important for nurses to assess stool consistency accurately. Also, non-physician personnel need to understand the testing criteria so as to be able to inform physicians about which tests should be performed at what timing for an accurate diagnosis. The present study therefore aimed to evaluate a multifaceted, multidisciplinary intervention for DS of CDI.

## Materials and methods

### Study setting

The present study was conducted at Tokyo Metropolitan Tama-Nambu Chiiki Hospital, a 26-department, 287-bed (high-care unit with 6 beds) community hospital with no infectious disease physicians on the staff. The study period included the period before the pandemic (2018/4–2020/3), during the pandemic (2020/4–2022/3), and after DS implementation (2022/4–2023/3). The impact of the DS for CDI during the pandemic on the CD testing rate and CDI incidence was analyzed. Nurses assessed stool consistency using the Bristol Stool Scale^15^ during the entire study period. Before the intervention, if an enzyme immunoassay (EIA) (TechLab, Blacksburg, VA, USA) returned antigen positive and toxin negative, a stool culture was performed, and the EIA was repeated.^16^ After the intervention, nucleic acid amplification assay (NAAT) (Beckman Coulter, California, USA) was performed if the EIA results returned antigen positive and toxin negative.

### Multifaceted interventions

The intervention comprised the following parts: (1) the addition of CD testing criteria to the hospital’s local guidelines; (2) provision of a tutorial on CD testing to physicians, nurses, and pharmacists; (3) assessment by clinical pharmacists and nurses of the need for CD testing in patients with nosocomial diarrhea and the issuance of recommendations for CD testing to physicians; (4) change in the assessment method from EIA to NAAT; and (5) provision of the data on the CD testing rate and CDI incidence in the hospital to healthcare workers every 3 months. The testing criteria included (1) diarrhea cases of unknown etiology >3 within 24 hours; (2) at least one case of diarrhea with abdominal cramps/pain; (3) frequency of increased diarrhea or worsening of abdominal pain within 24 hours >3 times that of the normal diarrhea frequency in patients with chronic diarrhea; (4) persistence of diarrhea >24 hours and increased fecal excretion, or greater abdominal pain or discomfort in patients with an enterocutaneous fistula. Excluding criteria were as follows: (1) starting a postprandial diet or laxatives within 48 hours and (2) CDI diagnosis within 7 days. The tutorial emphasized the introduction of the new testing criteria and the fact that CDI cannot be ruled out without testing.

### Definitions

CDI was diagnosed on the basis of the following signs and symptoms in addition to positive findings for toxins on an EIA or NAAT: (1) at least three cases of diarrhea with severity >6 on the Bristol Stool Scale within 24 hours, (2) at least one case of diarrhea with abdominal pain or tenderness,^17^ and (3) at least three cases of exacerbated diarrhea or abdominal pain within 24 hours in patients with chronic diarrhea. In patients with an intestinal fistula, the persistence of diarrhea for more than 24 hours, greater than usual fecal excretion, and severe abdominal pain or abdominal discomfort were considered indicative of CDI. Patients aged <2 years were excluded.^12^

A new case of CDI was defined by the absence of signs or symptoms within the previous 8 weeks and positivity for CD on an EIA or NAAT. A recurrent case was defined by a history of CDI positivity within 2–8 weeks of the initial infection, recurrence of signs and symptoms, and positive results on an EIA or NAAT.^17^

The following three epidemiological categories were also established: (1) healthcare facility-onset (HO) disease, in which a positive stool specimen was collected more than 3 calendar days after hospital admission; (2) community-onset healthcare facility-associated (CO-HCFA) disease, in which a positive stool specimen was collected in an outpatient setting or within 3 days after hospital admission in a patient with a documented, overnight stay in a healthcare facility (i.e., hospital or long-term care facility) in the 12 weeks prior to the collection of a positive stool specimen; and (3) community-associated (CA) disease, in which a positive stool specimen was collected in the outpatient setting or within 3 calendar days after hospital admission in a patient with no documented overnight stay in a healthcare facility in the 12 weeks prior to the collection of a positive stool specimen.^17^

CDI severity was rated as mild if serum creatinine (SCr) was <1.5 mg/dL and white blood cell (WBC) count was <15,000 cells/mL; severe if SCr was ≥1.5 mg/dL or WBC was ≥15,000 cells/mL; and fulminant if ileus, toxic megacolon, hypotension, or shock was present.^17^

### Outcomes

The primary outcome was the monthly CD testing rate and the CDI incidence per 10,000 PD. The secondary outcomes were the severity, classification, mortality rate, CDI recurrence, length of hospital stay, and test sensitivity before and after the intervention, with stool characteristics being used as an index of efficacy.

### Statistical analysis

The CD testing rate and CDI incidence were calculated monthly per 10,000 PD. Segmented regression in interrupted time-series analysis (ITSA) was performed before the pandemic (2018/4–2020/3), during the pandemic (2020/4–2022/3), and after the intervention (2022/4–2023/3). The ITSA value based on monthly intervals was 24 before the pandemic, 24 during the pandemic, and 12 after the multifaceted intervention period. The χ^2^ test was conducted before and after the intervention for patients with a Bristol Stool Scale ≤5 to assess the sensitivity of the testing methods. *P* < .05 was considered to indicate statistical significance. Stata version 17.0 (StataCorp, College Station, TX) was used for all statistical analyses. The institutional review board at Tokyo Metropolitan Tama-Nambu Chiiki Hospital approved this study, and patient consent was waived because the present study was a quality improvement study.

## Results

During the study period, the median CD testing rate per period (interquartile range [IQR]) was 27.4 (21.3–30.8), 29.9 (22.2–40.0), and 51.1 (41.0–57.5) per 10,000 PD for the period before the pandemic, during the pandemic, and after the implementation of the DS. The median CDI incidence (IQR) was 3.0 (1.5–5.3), 1.9 (0.0–2.7), and 5.9 (2.7–6.8) per 10,000 PD for the respective period.

**Figure 1.**
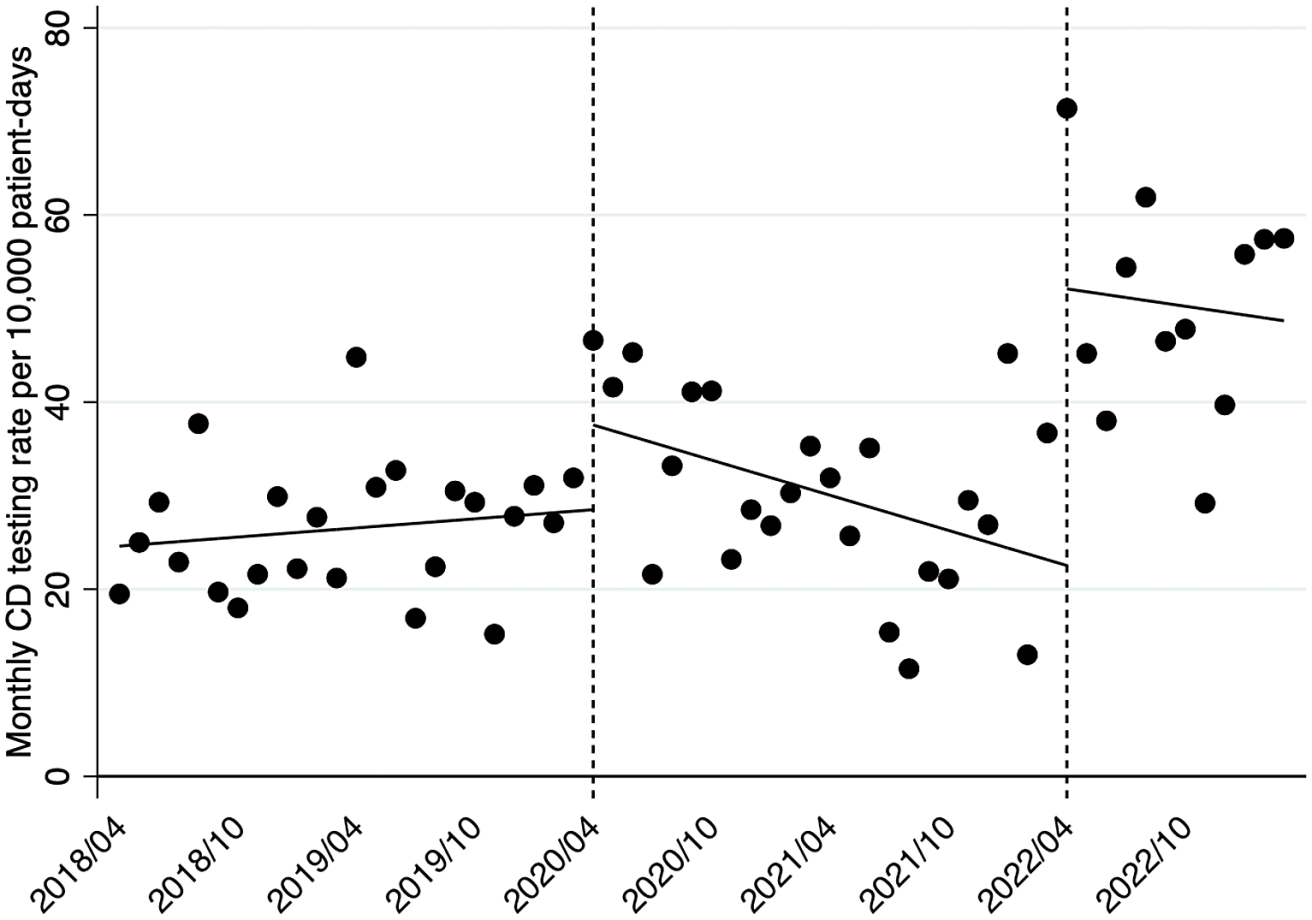
Changes in the monthly CD testing rate per 10,000 PD before the pandemic, during the pandemic, and after DS implementation. *Note*: CD, *Clostridioides difficile*; PD, patient-days; DS, diagnostic stewardship.

With respect to the CD testing rate, ITSA demonstrated an increasing trend before the pandemic (+0.16; 95% CI, −0.14–0.46; *P* = .28), but the level then increased immediately during the pandemic (+9.06; 95% CI, 0.91–17.21; *P* = .03) before decreasing (−0.79; 95% CI, −1.44–−0.13; *P* = .02). The level increased significantly immediately after the implementation of the intervention (+29.6; 95% CI, 14.94–44.21; *P* < .01), and the trend showed no change at this juncture (+0.31; −2.02–2.65; *P* = .79) (Figure 1).

**Figure 2.**
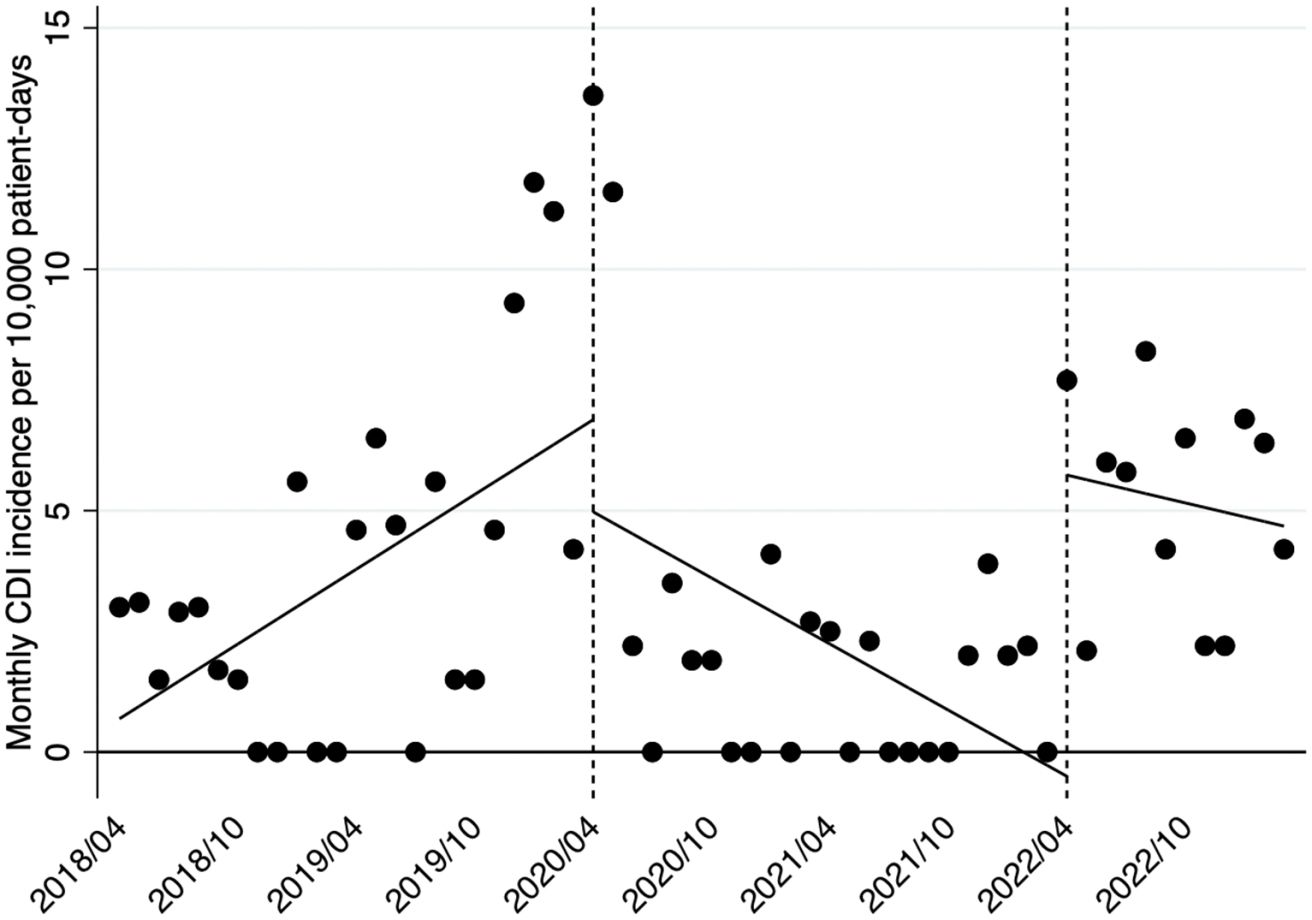
Changes in the monthly *Clostridioides difficile* infection incidence per 10,000 PD before the pandemic, during the pandemic, and after DS implementation. *Note*: PD, patient-days; DS, diagnostic stewardship.

With respect to the CDI incidence, ITSA demonstrated a statistically increasing trend (+0.26; 95% CI, 0.04–0.48; *P* = .02) as with the CD testing rate before the pandemic, but the level immediately fell during the pandemic (−1.91; 95% CI, −8.09–4.27; *P* = .54). The trend in the incidence decreased during the pandemic (−0.48; 95% CI, −0.85–−0.12; *P* = 0.01). The level increased significantly after the intervention (+6.24; 95% CI, 2.75–9.73; *P* < .01), and the trend showed no significant change (+0.13; 95% CI, −0.29–0.56; *P* = .53) (Figure 2). Table 1 shows the data in detail.

**Table 1.** Changes in the CD testing rate and CDI incidence before the pandemic, during the pandemic, and after DS implementation as analyzed by interrupted time-series analysis

Variable	Slope before pandemic	Regression intercept after pandemic	*P* value	Change in slope between period before and after pandemic	*P* value	Regression intercept after intervention	*P* value	Change in slope between period after pandemic and after DS implementation	*P* value
Monthly CD testing rate per 10,000 PD (95% CI)	0.16 (−0.14–0.46)	9.06 (0.91–17.21)	.03	−0.79 (−1.44–−0.13)	.02	29.6 (14.94–44.21)	<.01	0.32 (−2.02–2.65)	.79
Monthly CDI incidence per 10,000 PD (95% CI)	0.26 (0.04–0.48)	−1.91 (−8.09–4.27)	.54	−0.49 (−0.85–−0.12)	.01	6.24 (2.75–9.73)	<.01	0.13 (−0.29–0.56)	.53

Abbreviations: CD, *Clostridioides difficile*; CDI, *Clostridioides difficile* infection; DS, diagnostic stewardship; PD, patient-days.

With respect to individual CDI cases, 73.3%–74.1% were non-severe during the entire period, and HO-CDI and CO-HCFA-CDI accounted for 91.4%, 81.5%, 76.7% and 6.9%, 11.1%, 16.7% of the cases in the respective period. Table 2 shows the composition of the CDI patients during each period.

**Table 2.** Characteristics of the patients with CDI during the study period

Characteristics		Prepandemic	Postpandemic	Postintervention
		(2018/4–2020/3)	(2020/4–2022/3)	(2022/4–2023/3)
Number of patients with CDI		58	27	30
Median age (IQR)		83.4 (78.1–88.9)	81.9 (78.4–90.6)	85.6 (81.4–89.6)
Male sex (*n*, %)		35 (60.3)	15 (55.6)	14 (46.7)
Severity (*n*, %)	Non-severe	43 (74.1)	20 (74.1)	22 (73.3)
	Severe	12 (20.7)	5 (18.5)	8 (27.7)
	Fulminant	3 (5.2)	2 (7.4)	0 (0)
Classification (*n*, %)	Healthcare facility onset	53 (91.4)	22 (81.5)	23 (76.7)
	Community onset healthcare Facility associated	4 (6.9)	3 (11.1)	5 (16.7)
	Community associated	1 (1.7)	2 (7.4)	2 (6.7)
30-day mortality (*n*, %)		8(13.8)	8 (29.6)	5 (16.7)
Recurrence (*n*, %)		10 (17.2)	1 (3.7)	1 (3.3)
Length of hospital stay (median [IQR])		58.5(35.0–89.8)	28.0 (19.0–50.0)	32.0 (18.8–42.5)

Abbreviations: CDI, *Clostridioides difficile* infection; IQR, interquartile range.

Of the patients who underwent CD testing, the percentage of those with a Bristol Stool Scale ≤5 drastically changed from 148 of 736 (20.1%) in the preintervention period to 24 of 320 (7.5%) in the postintervention period (*P* < .01). A comparison of the EIA/Stool culture method with EIA/NAAT revealed a positivity rate of 34% (32/94) and 40% (14/35), respectively (*P* = .53).

## Discussion

The present study evaluated the impact of a multifaceted DS for CDI in a small, Japanese hospital during the COVID-19 pandemic. The CD testing rate and CDI incidence, which had been declining during the pandemic, increased immediately after the intervention and remained at the same level during the intervention. Moreover, it was deemed to have reduced overdiagnosis by obviating the need for testing in patients with a stool consistency not conforming to the stated criteria. The findings suggested that even in small hospitals, the implementation of multidisciplinary DS can be effective in optimizing the diagnosis of CDI.

Previous reports of DS for CDI have included a single intervention consisting of tutorials^18^ aimed at increasing the CD testing rate, but few studies have examined a DS that is both multidisciplinary and multifaceted. Appropriate and timely testing of the right patient^19^ is crucial for the optimal outcome, but this can be achieved only through the cooperation of the nursing staff in assisting physicians with determining the most appropriate timing for CD testing and of pharmacists in clarifying which patients require CD testing.

The underdiagnosis of CDI might be the result of a lack of awareness about the disease^20^ stemming from that fact that it has a lower incidence than other, healthcare-associated infections, such as Methicillin-resistant Staphylococcus aureus and extended-spectrum beta-lactamases-producing Enterobacterales infections. Therefore, in the present study, providing regular feedback may have helped to maintain awareness of the CDI risk among many healthcare workers. The CD testing rate reportedly correlates with the CDI incidence.^21^ Similarly, the CDI incidence in the present study increased immediately following the increase in the CD testing rate as part of the intervention. Furthermore, maintaining the CD testing rate postintervention stabilized the CDI incidence rate. Moreover, after the intervention, CO-HCFA-CDI increased while HO-CDI decreased possibly because previous tests were performed and the diagnosis was made after several days’ hospitalization, resulting in the categorization of more cases as HO-CDI. However, postintervention, tests began to be performed at an earlier stage, leading to the more accurate categorization of cases as CO-HCFA-CDI together with a corresponding decrease in HO-CDI cases. Appropriate timing of testing can improve patient outcomes as well as infection prevention and control by reducing nosocomial transmission.

The quality of stool specimens is important for testing. A previous study describing a DS for CDI based on a clinical decision support system aimed at reducing excessive testing^22^ excluded solid stool specimens from the testing criteria.^23^ On the other hand, on the assumption that the critical issue was unawareness of the importance of CDI diagnostic testing rather than the qualities of the stool specimen, the present study did not restrict testing. Nonetheless, the quality of stool samples collected from the patients in the present study improved following the implementation of the intervention. Thus, raising the awareness of medical staff in the various, relevant disciplines may not only improve awareness of the value of testing but may also contribute to improving the accuracy of stool sample assessments.

The present study has several limitations. Although it successfully implemented a multidisciplinary, multifaceted DS for CDI, its findings may not be generalizable because the study was monocentric, quasi-experimental, and of relatively short duration. Further research at multiple, independent centers is needed to verify the present findings. Moreover, the use of laxatives was not determined in patients receiving the tests. Thus, overdiagnosis may have resulted. However, the data showed an improvement in stool characteristics following the intervention. Furthermore, the change in the recommended antimicrobial therapy from metronidazole to vancomycin and fidaxomicin may have affected the recurrence rate. Also, the testing method was also changed from EIA to NAAT after the intervention; therefore, the latter’s higher sensitivity cannot be ruled out as a reason for the observed increase in CDI incidence. However, because the CD testing rate increased, the change in the testing method may have had little effect on increasing the CDI incidence. The optimal frequency of testing was unable to be determined in the present study. Finally, goodness of fit or lags related to the differences caused by the changes made in the study were not assessed.

## Conclusion

To optimize CD testing, a multidisciplinary, multifaceted DS may be effective for countering underdiagnosis and may contribute to improving the accuracy of stool sample assessments. The prevalence of infections caused by resistant bacteria is on the rise,^24^ and inappropriate antimicrobial use increases this risk, including that of CDI. Hospital infection prevention and control measures, continuous symptom surveillance, and education of healthcare workers are vital to enabling the early detection of symptoms by allowing correct diagnosis, appropriate, individualized treatment, and prevention of nosocomial CD transmission.
